# Spatial Variations in Microbial Community Composition in Surface Seawater from the Ultra-Oligotrophic Center to Rim of the South Pacific Gyre

**DOI:** 10.1371/journal.pone.0055148

**Published:** 2013-02-06

**Authors:** Qi Yin, Bingbing Fu, Bingyu Li, Xiaochong Shi, Fumio Inagaki, Xiao-Hua Zhang

**Affiliations:** 1 College of Marine Life Sciences, Ocean University of China, Qingdao, People’s Republic of China; 2 Kochi Institute for Core Sample Research, Japan Agency for Marine-Earth Science and Technology, Nankoku, Kochi, Japan; University of New South Wales, Australia

## Abstract

Surface seawater in the South Pacific Gyre (SPG) is one of the cleanest oceanic environments on earth, and the photosynthetic primary production is extremely low. Despite the ecological significance of the largest aquatic desert on our planet, microbial community composition in the ultra-oligotrophic seawater remain largely unknown. In this study, we collected surface seawater along a southern transect of the SPG during the Integrated Ocean Drilling Program (IODP) Expedition 329. Samples from four distinct sites (Sites U1368, U1369, U1370 and U1371) were examined, representing ∼5400 kilometers of transect line from the gyre heart to the edge area. Real-time PCR analysis showed 16S rRNA gene abundance in the gyre seawater, ranging from 5.96×10^5^ to 2.55×10^6^ copies ml^−1^ for *Bacteria* and 1.17×10^3^ to 1.90×10^4^ copies ml^−1^ for *Archaea*. The results obtained by statistic analyses of 16S rRNA gene clone libraries revealed the community composition in the southern SPG area: diversity richness estimators in the gyre center (Sites U1368 & U1369) are generally lower than those at sites in the gyre edge (Sites U1370 & U1371) and their community structures are clearly distinguishable. Phylogenetic analysis showed the predominance of *Proteobacteria* (especially *Alphaproteobacteria*) and *Cyanobacteria* in bacterial 16S rRNA gene clone libraries, whereas phylotypes of *Betaproteobacteria* were only detected in the central gyre. Archaeal 16S rRNA genes in the clone libraries were predominated by the sequences of Marine Group II within the *Euryarchaeota*, and the *Crenarchaeota* sequences were rarely detected, which is consistent with the real-time PCR data (only 9.9 to 22.1 copies ml^−1^). We also performed cultivation of heterotrophic microbes onboard, resulting in 18.9% of phylogenetically distinct bacterial isolates at least at the species level. Our results suggest that the distribution and diversity of microbial communities in the SPG surface seawater are closely related to the ultra-oligotrophic oceanographic features in the Pacific Ocean.

## Introduction

The South Pacific Gyre (SPG) is the largest gyre of the world and extends a long way from the Pacific’s swell in the east (20°S) to the oceanic crust of the Cretaceous period in the west (45°S). Due to its isolated location, the center of the gyre is scarcely influenced by nutritional influx and pollution from the continents [1]. Satellite imaging has shown that the SPG has the lowest surface chlorophyll-*a* (Chl-*a*) concentrations of the worlds’ oceans (<0.02 µg l^−1^) [2] and UV absorption measurements have demonstrated that it is the clearest water in the world [3]. The area of the low-chlorophyll (≤0.14 µg of Chl-*a* l^−1^) region (5.2×107 km^2^) in the SPG is more than two times larger than that of North America [4]. Because of its size and low primary productivity indicated by surface Chl-*a* concentration, this ultra-oligotrophic ocean region has been considered the largest oceanic desert on our planet [1]. All these physical and geochemical parameters make the SPG a unique and extreme habitat of life, in contrast to other marine ecosystems. Studies on the abundance of facultatively aerobic anoxygenic phototrophic bacteria (AAPB) and the diversity of bacterial groups performing N_2_-fixation in the SPG were published recently [5], [6]. However, distribution, phylogenetic diversity and structure of the microbial communities in this ultra-oligotrophic ocean remain largely unkown.

In this study, we report biogeographical distribution of bacterial and archaeal communities in surface seawater along a southern transect of the SPG using molecular ecological and cultivation-based analyses. By comparing abundance, phylogenetic diversity and community structure at four distinct locations in the SPG, we discuss the relationship between microbial communities and oceanographic features of the SPG.

## Materials and Methods

### Ethics Statement

No specific permits were required for the described field studies. The location is not privately-owned or protected in any way, and the field studies did not involve endangered or protected species.

### Surface Seawater Sampling and Environmental Factors

**Figure 1:**
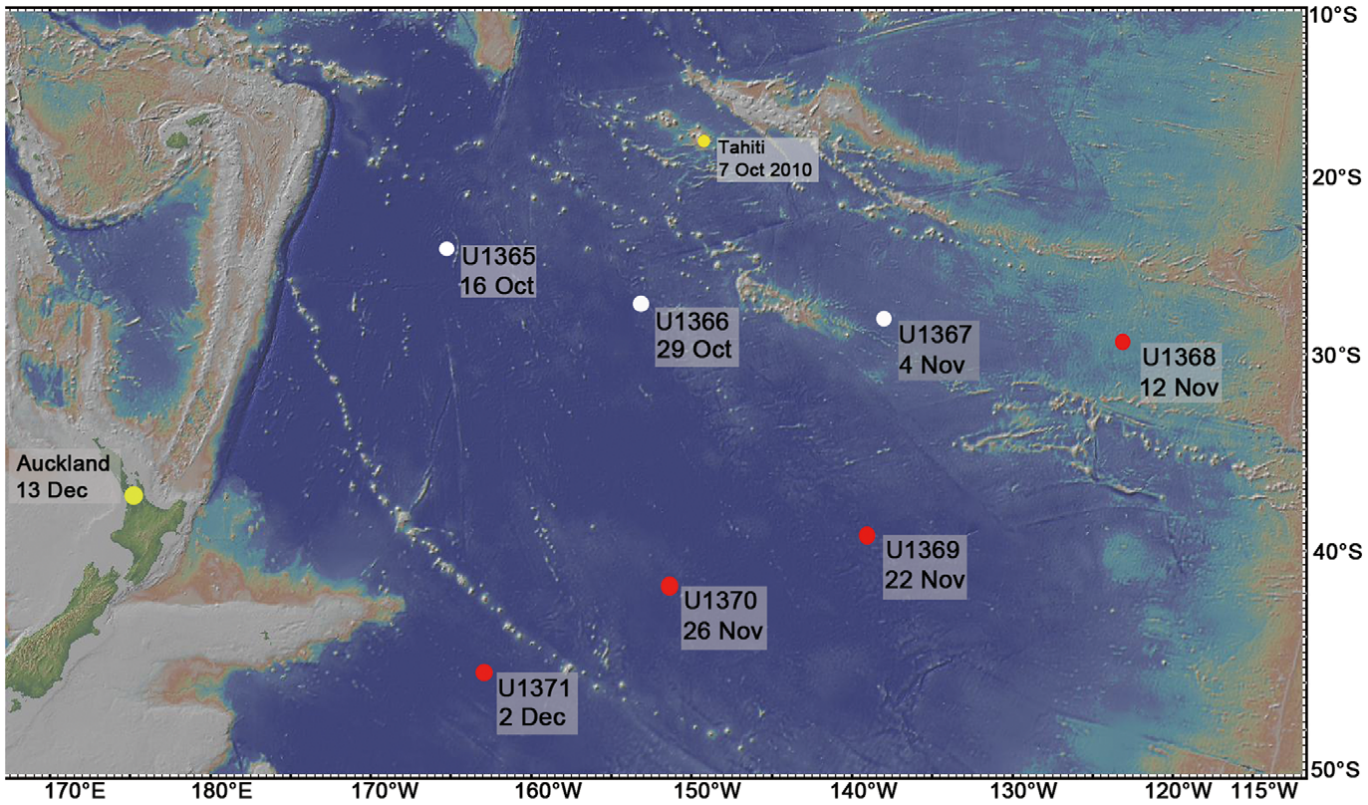
The map of SPG stations. The nine dots represented the route of IODP329, and the red dots represented the stations sampled in this study.

Seawater samples were obtained onboard *the drilling research vessel JOIDES Resolution* during the Integrated Ocean Drilling Program (IODP) Expedition 329, from 9 October to 13 December 2010. Four sites located along the transect line from the center to the southern edge of the SPG were selected for this study (Figure 1, Table 1). Site U1368 was located in the center of the gyre, whereas Site U1369 and Site U1370 were located midway between the center and the southern edge of the gyre. Site U1371 was located in the upwelling region just south of the gyre.

**Table 1:** Environmental factors of the four sites. Almost all nutrients concentration increased from the gyre center to the edge, and many concentrations close to the detection limit [6].

Site	Date	Time	Location	Tem [°C ]	Salinity at 80 m(psu) [48]	Chl a [µg l^−1^][6]	Nitrate [µM] [6]	Ammonium [µM] [6]	Phosphate [µM] [6]
U1368	12 Nov	14∶30 pm	27°55′S, 123°10′W	22	35.26	∼0.02	∼0.5	∼0.02	∼0.05
U1369	22 Nov	14∶45 pm	39°19′S, 139°48′W	17.5	34.33	∼0.30	∼1.5	∼0.12	∼0.6
U1370	26 Nov	16∶00 pm	41°51′S, 153°06′W	15	34.45	∼0.35	∼1.2	∼0.12	∼0.8
U1371	2 Dec	9∶00 am	45°58′S, 163°11′W	12	34.70	∼0.40	∼3.8	∼0.18	∼0.9

Surface seawater samples at the water depth of ∼1 meter were collected with acid-washed, sterile (121°C/15 min) 500 ml glass bottles immediately after arrival at the sites to avoid influence from ship activities. The seawater samples were immediately transported to the shipboard microbiology laboratory, and filtered through 0.22 µm Whatman Nucleopore Track-Etch Membranes (Whatman Schleicher & Schuell, Keene, NH, USA) under aseptic conditions. The membranes were stored at −80°C until the shore-based molecular analysis. Additional seawater samples were stored at 4°C for bacterial cultivations, which was initiated onboard within 1 hour of sampling as described below.

### Bacterial Isolation

Aerobic heterotrophic bacteria were cultured on Marine Agar (Difco) and Marine R2A agar (Fluka R2A agar was prepared with seawater instead of distilled water). The plates were incubated at 25°C for 7 days. Individual colonies were randomly picked and purified by streaking three times on fresh medium. Stocks were preserved at −80°C in sterile 0.9% (w/v) NaCl supplemented with 15% (v/v) glycerol. Genomic DNA of the isolates was extracted by phenol/chloroform extraction and 16S rRNA genes were amplified and sequenced to determine the phylogenetic characteristics.

### DNA Extraction and PCR Amplification

Sample membranes were cut into small pieces and put into sterile 1.5 ml tubes. Then, 400 µl of STE buffer (100 mM NaCl, 10 mM Tris-HCl, 1 mM EDTA, pH 8.0) and 80 µl of 10% (w/v) sodium dodecyl sulfate (SDS) were added into each tube. After incubation at 65°C for 20 min, each tube was vortexed and then centrifuged at 1400 *g* for 15 min [7]. The supernatant was transferred into new tubes, extracted with equal amounts of phenol, and further extracted twice with equal amounts chloroform–isoamyl alcohol (24∶1). The final aqueous layer was recovered, mixed with two volumes of absolute ethyl alcohol and 0.1 volume of 3 M sodium acetate (pH 5.2) for precipitation, and re-suspension in 40–50 µl of sterile double distilled water (pH 8.0).

16S rRNA genes were amplified by PCR using bacterial primers 8F (5′-AGAGTTTGATCCTGGCTCAG-3′) and 1492R (5′-GGTTACCTTGTTACGACTT-3′) [8], and archaeal primers Arch21F (5′-TTCCGGTTGATCCYGCCGGA-3′) and Arch958R (5′-YCCGGCGTTGAMTCCAATT-3′) [9]. In order to minimize the effect of possible heteroduplex formation, a re-conditioning PCR procedure [10] was used, in which three extra rounds of thermal cycling were performed in a fresh reaction mix with 5 µl amplicon from the original PCR used as template. The 50 µl PCR reactions included 200 µM of each dNTP, 1.5 mM MgCl_2_, 0.5 µM of each primer, 0.02% (w/v) Bovine serum albumin (BSA), 0.05 U *Taq* polymerase (Fermentas International Inc), and 5 µl 10 × *Taq* buffer with KCl. The thermal cycling conditions for the bacterial PCR included a touchdown series, in which the annealing temperature decreased from 65°C to 55°C by 1°C per cycle, followed by 15 cycles at 55°C. Each cycle started with a 1 min denaturation step at 95°C, continued with 1 min annealing, and ended with an extension step of 2.5 min at 72°C. An initial denaturing step at 95°C for 5 min and a final extension step of 10 min at 72°C was used. The archaeal PCR cycling conditions consisted of 30 cycles of denaturing at 95°C for 1 min, annealing at 55°C for 1 min and extension at 72°C for 1 min.

### Construction of Clone Libraries and Sequencing

PCR products of the 16S rRNA gene from surface seawater samples were purified and inserted into pMD18-T vectors (TaKaRa Co., Dalian, China), and *Escherichia coli* TOP10 competent cells were used as transformers. Transformants were selected using Xgal-IPTG LB plates with 100 µg ml^−1^ ampicillin. Three hundred white colonies were picked from a single plate for each library and PCR amplified with vector specific primers M13f (5′-GTAAAACGACGGCCAG-3′) and M13r (5′-GTTTTCCCAGTCACGAC-3′) for checking correct insertion. Two hundred bacterial and 100 archaeal clones were finally bidirectionally sequenced. Chimeric sequences were detected and excluded from further analysis by use of the CHECK_CHIMERA program of the Ribosomal Database Project II (RDP-II,http://rdp.cme.msu.edu/html/) [11].

### Phylogenetic and Statistical Analyses

Closest relatives to the 16S rRNA gene sequences were searched for in GenBank [12] by use of the BLASTn program [13]. 16S rRNA gene sequences from all the clones were aligned with the ClustalX program (version 2.0) [14], and grouped into operational taxonomic units (OTUs) based on 3% (*Bacteria*) and 2% (*Archaea*) dissimilarity cut-off values calculated by the DOTUR program [15]. We also used DOTUR program to calculate diversity indices including coverage, species evenness (*J*), abundance-based coverage estimator ACE [16], species richness estimator Chao1 [17], Shannon–Wiener index (*H*), and Shannon index (*D*) [18]. The coverage was calculated as *C* = [1 – (*n_1_/N*) ×100], where *n_1_* represents the OTUs represented by only one clone (i.e. singleton) and N represents the total number of clones in a library. It presents the probability that in the given library all the unique sequences were detected at least once [19]. UNIFRAC principal coordinate analysis (PCoA) of weighted sequence data following previously established procedures [20] were carried out to statistically determine the relationship between the bacterial and archaeal communities at the four sampling sites. Correlation between microbial community structure and environmental factors among the four sites were analyzed by canonical correspondence analysis (CCA) using the software Canoco (version 4.5, Microcomputer Power) [21].

Phylogenetic trees were constructed using the Kimura 2-parameters distance matrix [22] and the neighbor-joining algorithm [23] by use of the PHYLIP package (version 3.69). A random selection clone sequence for each OTU was included. Phylogenetic affiliation of bacterial clones was determined by use of the RDP-II classifier [24].

### Quantification of Major Microbial Groups

The major microbial groups in each sample were quantified by real-time PCR, with three independent experimental replicates, including total *Bacteria*, total *Archaea*, *Alphaproteobacteria*, *Betaproteobacteria*, *Actinobacteria*, *Bacteroidetes*, *Firmicutes*, *Cyanobacteria* and *Crenarchaeota*. The chosen primers and corresponding annealing temperatures are shown in Table 2 according to previously established real-time PCR protocols [25], [26]. All real-time PCR assays were conducted in triplicate using ABI Prism 7500 Sequence Detection System (Applied Biosystems, Foster City, CA, USA). Each 20 µl real-time PCR reaction contained the following components: 10 µl SYBR Green Realtime PCR Master Mix (TaKaRa, Tokyo, Japan), 1 µl of each primer (10 µM), 6 µl H_2_O, and 2 µl 96 fold diluted template DNA (according to the results of our preliminary experiment). To determine the relationship between the PCR cycle threshold (Ct) value and copy number, standard curves were obtained by using vector plasmids containing the 16S rRNA gene of a representative of each target group. Briefly, plasmid DNA was extracted with Mini Plasmid Kit (Qiagen, Valencia, CA, USA), and quantified using PicoGreen and a Modulus Single Tube Multimode Reader fluorometer (Biochrom Ltd, Cambridge, UK). Then the extracted plasmid DNA was 10-fold serially diluted. Standard curve generated using plasmids to relate Ct value to gene copy number revealed linearity (R^2^ = 0.985) over several orders of magnitude of the plasmid DNA concentrations. Triplicates were performed in each order. The obtained high correlation coefficient (0.97) of the standard curves made comparison of the abundance of different genes reliable. The generated Ct value of each microbial group from each site was then recalculated as 16S rRNA gene abundance per milliliter of each sample. The PCR amplification efficiency was estimated to be 98.8%. One-way ANOVA followed by the Turkey test in the post-hoc analysis was performed by the SPSS v16.0 software to determine whether the microbial community structure at four sites had statistical distinction, and only the *P* value less than 0.05 was considered as significant difference.

**Table 2:** Primers for real-time PCR.

Target group	AnnealingTemp (°C)	Primer name and sequence (5′–3′ direction)	Reference
All bacteria	53	Eub338F: ACT CCT ACG GGA GGC AGC AG	[8]
		Eub518R: ATT ACC GCG GCT GCT GG	[49]
*Alphaproteobacteria*	60	Eub338F: ACT CCT ACG GGA GGC AGC AG	[8]
		Alf685R: TCT ACG RAT TTC ACC YCT AC	[8]
*Betaproteobacteria*	60	Eub338F: ACT CCT ACG GGA GGC AGC AG	[8]
		Bet680R: TCA CTG CTA CAC GYG	[50]
*Actinobacteria*	60	Actino235F: CGC GGC CTA TCA GCT TGT TG	[51]
		Eub518R: ATT ACC GCG GCT GCT GG	[49]
*Bacteroidetes*	60	Cfb319F: GTA CTG AGA CAC GGA CCA	[52]
		Eub518R: ATT ACC GCG GCT GCT GG	[49]
*Firmicutes*	60	Lgc353F: GCA GTA GGG AAT CTT CCG	[53]
		Eub518R: ATT ACC GCG GCT GCT GG	[49]
*Cyanobacteria*	60	Cya106F: CGG ACG GGT GAG TAA CGC GTG A	[54]
		Cya359R: CCC ATT GCG GAA RAT TCC CC	[54]
*Archaea*	56	Arch16F: CTG GTT GAT CCT GCC AG	[55]
		Arch344R: TTC GCG CCT GST GCR CCC CG	[56]
*Crenarcheota*	56	ArchGI334F: AGA TGG GTA CTG AGA CAC GGA C	[25]
		ArchGI554R: CTG TAG GCC CAA TAA TCA TCC T	[25]

### Nucleotide Sequence Accession Numbers

The sequences of 757 bacterial clones, 355 archaeal clones and 74 cultivated bacterial strains have been deposited in the GenBank database under the accession numbers JN985906 to JN986571, and JQ181802 to JQ182050, and JQ082124 to JQ082197, respectively.

## Results

### Environmental Factors

As shown in Table 1, the Chl-*a* and nutrient salts (nitrate, ammonium and phosphate) concentrations in the surface seawater of SPG were very low, and increased gradually from the gyre center to the edge.

### Diversity of Cultivated Bacteria

**Figure 2:**
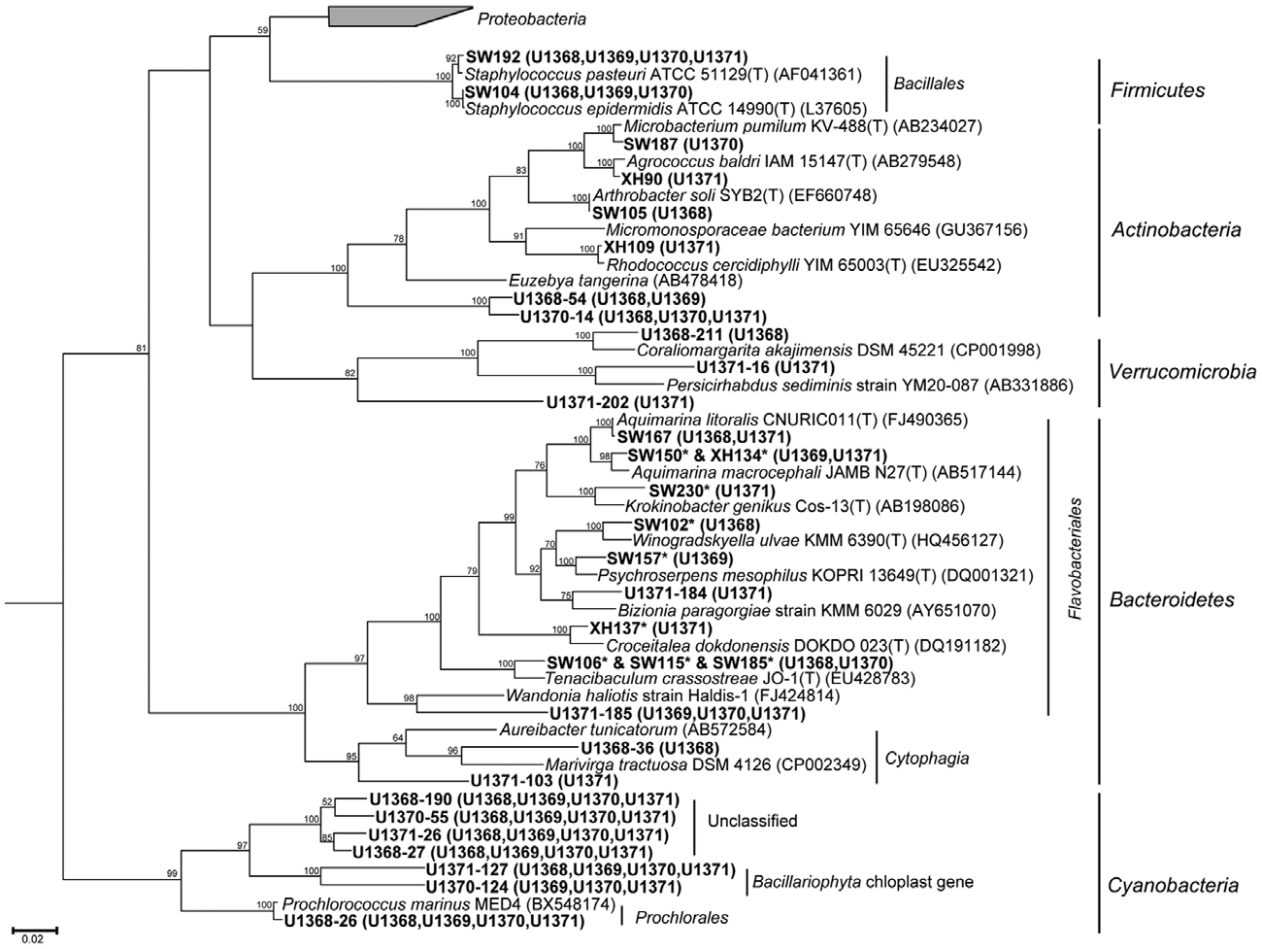
Phylogenetic tree of the 45 representative OTUs from bacterial 16S rRNA gene clone libraries (could represented all OTUs) and 32 cultivated bacteria from the four stations, constructed using neighbor-joining method of Phylip 3.66 based on the blast results of RDP classifer and EzTaxon server 2.1. The * represents the bacterial strains which were potential novel bacterial species. The phylogenetic neighbours were from the database of type strains with validly published prokaryotic names in the EzTaxon server (http://www.eztaxon.org/).

**Figure 3:**
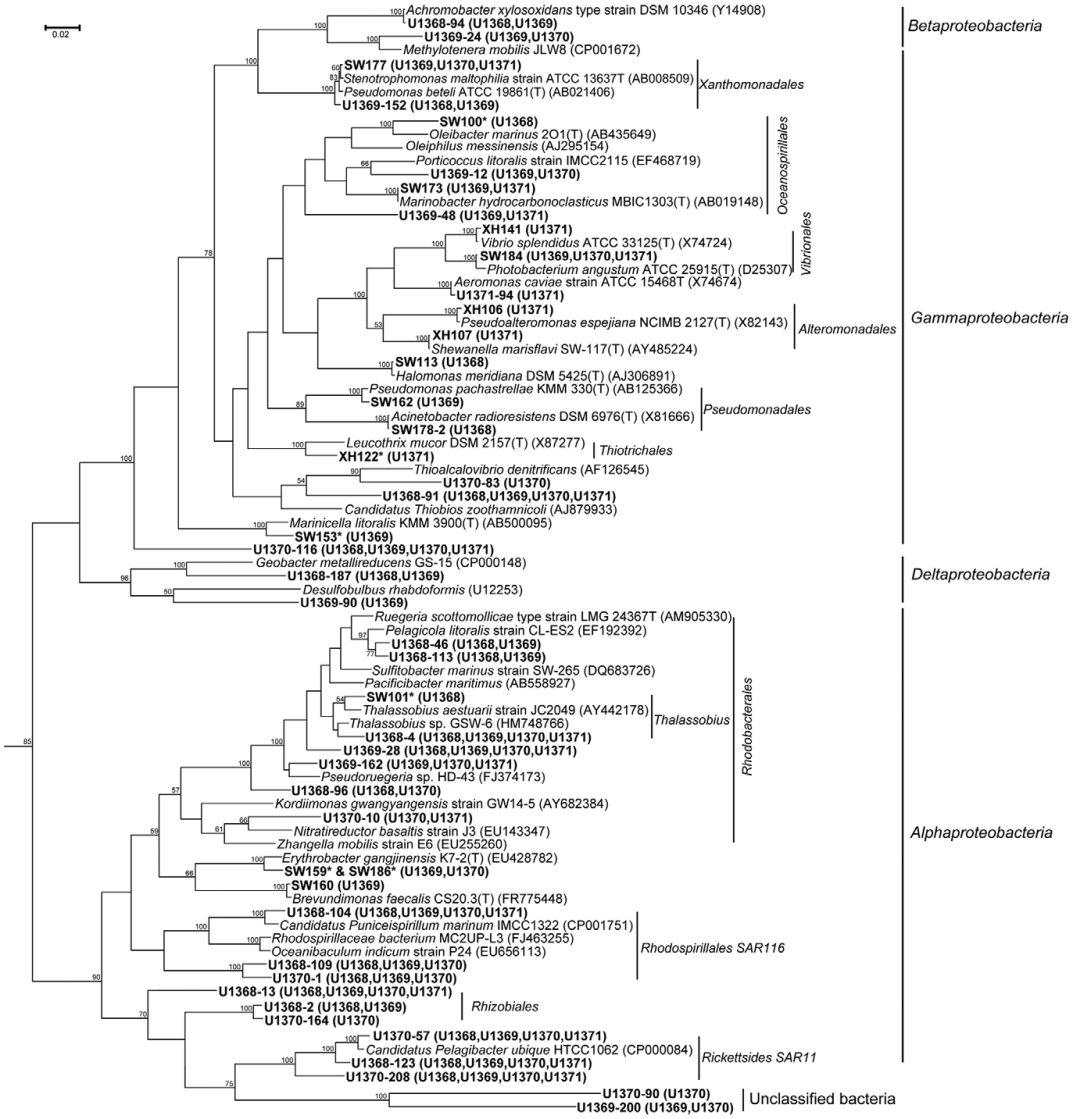
Phylogenetic tree of *Proteobacteria* and unclassified sections recovered from the 16S rRNA gene clone libraries.

The cultivated bacteria were mainly affiliated with the *Gammaproteobacteria*, accounting for 58.1%, followed by *Bacteroidetes* (16.2%), *Firmicutes* (13.5%), *Alphaproteobacteria* (6.8%) and *Actinobacteria* (5.4%). According to the results of BLASTn in the GenBank and the standard bacterial library of Korea (EzTaxon server 2.1), as high as 18.9% of bacterial strains (14 strains) were identified as potential novel species, with 16S rRNA gene similarities ≤97%. Among the 14 potential novel bacterial strains, SW100, SW102, SW115, SW185, SW230 and XH122 were distantly (≤94%) related to sequences of type species. *Photobacterium* was the dominant group in the members of *Gammaproteobacteria* (Figure 2 and Figure 3), accounting for 41.9% in *Gammaproteobacteria* and 24.3% in all the cultivated bacteria. Bacterial clone libraries and cultivated bacteria shared four orders: *Xanthomonadales*, *Oceanospirillales*, *Rhodobacterales* and *Flavobacteriales*.

### Diversity of Bacterial 16S rRNA Gene Clone Libraries

In total, 757 bacterial 16S rRNA gene sequences (>1400 bp long) were analyzed. The clones comprised 661 unique sequences distributed over 122 OTUs at 3% cutoff value and four clone libraries only shared 15 common OTUs. The diversity metrics, including Chao1 and ACE estimators, indicated that diversity in the environment was generally low (Table 3).

**Table 3:** The diversity indices and richness estimators of the bacterial and archaeal 16S rRNA gene clone libraries. OTUs of the 16S rRNA gene sequences were determined at 3% for bacteria and 2% for archaea sequence distance cut-off using the DOTUR program.

Clone library	No. of clone	No. of singleton	No. of OTUs	*C*	*H*	*1/D*	*J*	S_ACE_	S_chao1_
BU1368	180	166	39	88.9	4.37	15.48	0.828	71.0	63.4
BU1369	196	155	54	85.7	4.80	16.10	0.834	91.3	86.5
BU1370	196	193	64	79.6	5.19	23.53	0.865	128.6	142.8
BU1371	185	166	61	81.1	5.06	18.44	0.853	115.9	101.6
AU1368	88	66	13	92.0	2.40	3.53	0.650	28.2	34
AU1369	92	72	20	83.7	2.96	4.97	0.683	69.8	111
AU1370	88	63	17	89.8	2.66	3.69	0.649	29.5	29
AU1371	87	60	15	90.8	2.23	2.52	0.571	28.5	29

The coverage (*C),* Shannon-Weiner (*H), Simpson (D)* and *evenness (J) indices,* and *S_ACE_* and *S_chao1_* richness estimators were calculated using the OTU data.

After the BLASTn analysis in GenBank, 87.7% of the OTUs showed high identity (≥97%) with known nucleic acid sequences, ∼7% were distantly (≤95%) related to the known sequences, and three OTUs has low similarity (<90%) with the known sequences.

**Figure 4:**
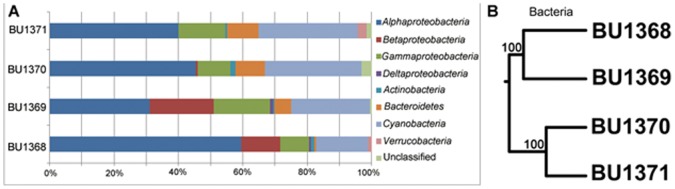
Relative abundance and clustering relationship of bacterial reads from the clone libraries of the surface seawater of SPG. A: Relative abundance of bacterial reads classified at the phylum level. B: UniFrac clustering relationship estimated using bacterial OTU representative clones. The numbers at the nodes are the jackknife bootstrap value calculated by UNIFRAC.

Of the 757 clones, 750 clones were affiliated with 5 bacterial phyla, while the remaining 7 clones were related to algal mitochondrion or chloroplast 16S rRNA gene sequences (Table S1). The 5 bacterial phyla included *Actinobacteria*, *Bacteroidetes*, *Cyanobacteria*, *Proteobacteria* and *Verrucomicrobia*. *Proteobacteria* was the most dominant group in our 16S rRNA gene clone libraries, accounting for 65.1% of the total 757 clones and 45.1% of the total 122 OTUs. Among the phylum *Proteobacteria*, *Alphaproteobacteria* constituted the most dominant group (43.7% of the total clones), followed by *Gammaproteobacteria* (12.8%), *Betaproteobacteria* (8.2%) and *Deltaproteobacteria* (0.4%) (Figure 4). *Cyanobacteria* was the second dominant and diverse group, accounting for 25.5% of the total clones and 26.2% of the total OTUs. *Bacteroidetes* accounted for 6.6% of the total clones and 17.2% of the total OTUs. According to clone libraries, compositions of three major bacterial groups showed obvious variation trends from the center to the gyre, with *Cyanobacteria* and *Bacteroidetes* increased and *Betaproteobacteria* decreased.

### *Proteobacteria*

*Alphaproteobacteria* was the most predominant group in the four bacterial 16S rRNA gene clone libraries (Figure 3 and Figure 4A). Thirty-four OTUs (331 clones) belonged to this group, with the percentage of 27.9% in the total OTUs. However, the diversity was low, only including the orders *Rhodospirillales* (3 OTUs, 12 clones), *Rhodobacterales* (8 OTUs, 75 clones), *Rickettsiales* SAR11 (11 OTUs, 140 clones), *Rhizobiales* (3 OTUs, 10 clones), and some unclassified *Alphaproteobacteria* (2 OTUs, 18 clones).

*Gammaproteobacteria* consisted of 97 clones (8.9%–17.3%, across the four libraries), including the orders *Xanthomonadales* (2 OTUs, 27 clones), *Chromatiales* (2 OTUs, 3 clones), *Aeromonadales* (1 OTUs, 7 clones) and many unclassified *Gammaproteobacteria* (13 OTUs, 60 clones). *Chromatiales* only appeared in BU1370 and *Aeromonadales* only appeared in BU1371, while *Xanthomonadales* only appeared in the center of the gyre (6 in BU1368 and 21 in BU1369).

*Betaproteobacteira* was a small group and was almost only detected in the center sites (U1368 and U1369) of the gyre, affiliating with *Burkholderiales* (1 OTUs, 60 clones) and *Methylophilales* (1 OTUs, 2 clones), which accounted for 12.2% of OTUs in BU1368, 19.9% OTUs in BU1369 and only 0.5% OTUs in BU1370. At the genus level, *Achromobacter* (99.5% similarity to *Achromobacter xylosoxidans*) was the dominant genus of the class (96.8%) (Table S1). There were 2 OTUs (3 clones, only in BU1368 and BU1369) that belonged to *Deltaproteobacteria*.

### *Cyanobacteria*

*Cyanobacteria* was the second most abundant group in all the four stations, accounting for 32 OTUs (26.3%) and 193 clones (25.5%). Twenty-four clones (12.4%) in total were affiliated with *Prochlorococcus*, while others were unclassified *Cyanobacteria*.

### *Bacteroidetes, Actinobacteria, Verrucomicrobia* and Unclassified Bacteria

Nineteen OTUs (47 clones) were affiliated with the *Bacteroidetes*, accounting for 15.6% in total OTUs and 6.2% in total clones. Most of them were unclassified, while only 2% belonged to the order *Cytophagales* and 27.2% belonged to the order *Flavobacteriales*. Only 7 clones were affiliated with the *Actinobacteria*, belong to unclassified branches. Seven *Verrucomicrobia* clones belong to the order *Puniceicocales* and *Verrucomicrobiales*. In addition, 7 unclassified clones were related to mitochondrion 16S rRNA gene sequences of algae.

### Diversity of Archaeal 16S rRNA Gene Clone Libraries

In total, 355 archaeal 16S rRNA gene clones were obtained from four clone libraries, representing 234 unique sequences and 38 OTUs with 2% cutoff value. According to the S_ACE_ and S_chao1_, U1369 has the highest archaeal diversity (S_ACE_ = 69.8, S_chao1_ = 111), but the diversity indexes were low as compared to Bacteria (Table 3).

**Figure 5:**
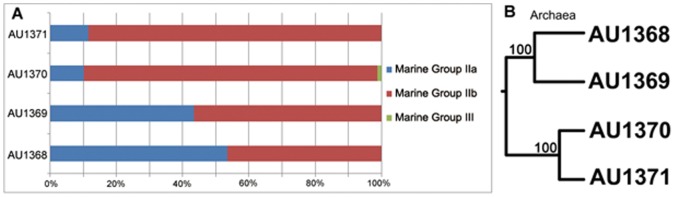
Relative abundance and clustering relationship of archaeal reads from the clone libraries of the surface seawater of SPG. A: Relative abundance of archaeal reads classified at the phylum level. B: UniFrac clustering relationship estimated using archaeal OTU representative clones. The numbers at the nodes are the jackknife bootstrap value calculated by UNIFRAC.

**Figure 6:**
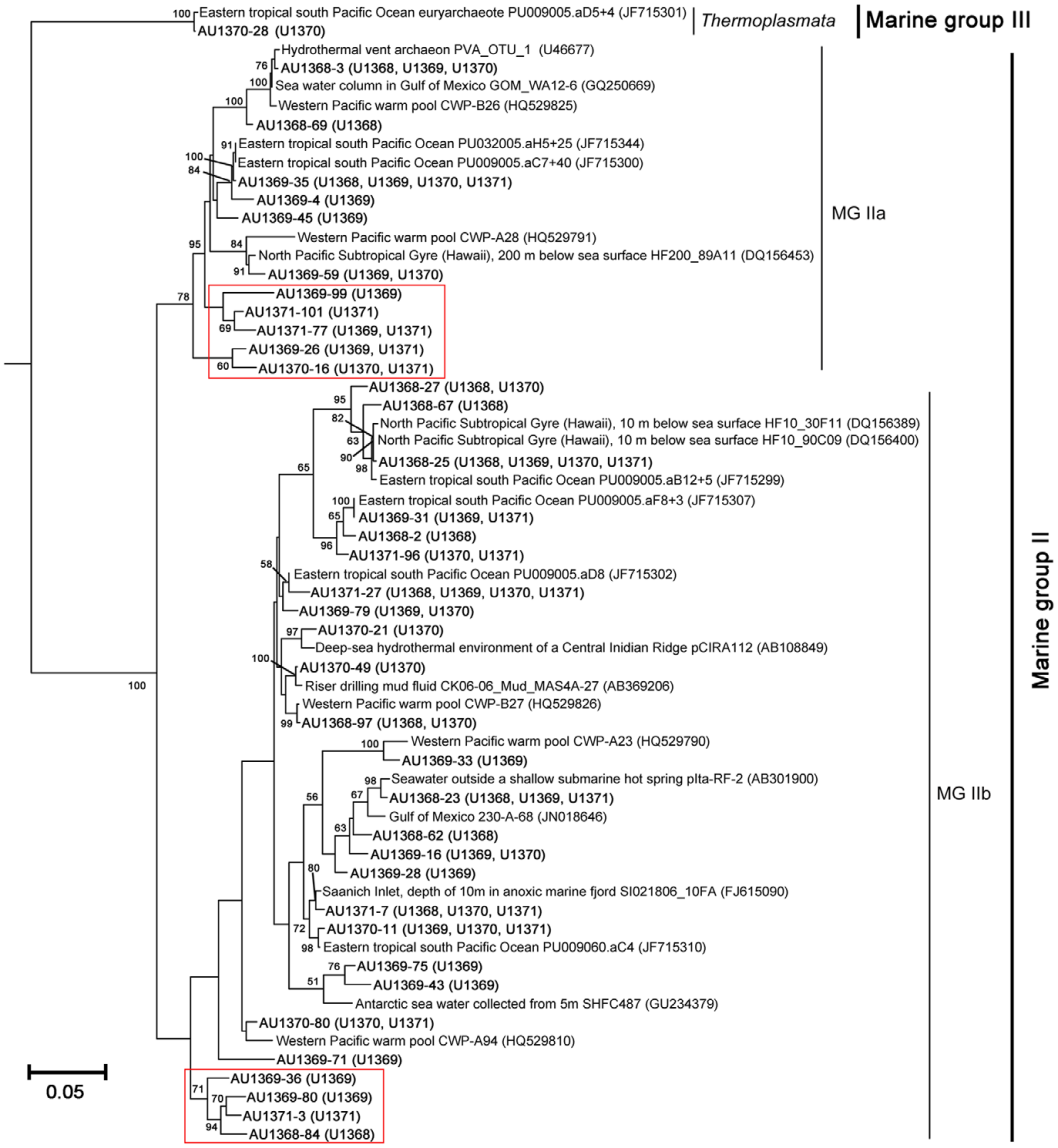
Phylogenetic tree of the archaeal sequences recovered from the 16S rRNA gene clone libraries, constructed using neighbor-joining method of Phylip 3.66. There were 38 OTUs, only one clone belonged to Marine Group III, while others were Marine Group II. The red passphrases represented the potential novel clades in *Euryarchaeota*.

All the archaeal clones were affiliated with the *Euryarchaeota*, in which almost all of them were Marine Group II, except OTU AU1370-28 (only 1 clone, Marine Group III), and they belong to three lineages: Marine Group IIa (MG IIb), Marine Group IIb (MG IIb) and *Thermoplasmata* (Figure 5A, Figure 6 and Table S2). Based on the BLASTn results, most (∼75%) of the clones were closely related with the archaeal clones obtained from permanent oxygen minimum zone of the eastern tropical South Pacific (95–99% similarities).

### Real-time PCR Analysis

To quantify the major microbial groups in the surface seawater of the SPG, we used the group-specific primers sets for real-time PCR analysis.

**Figure 7:**
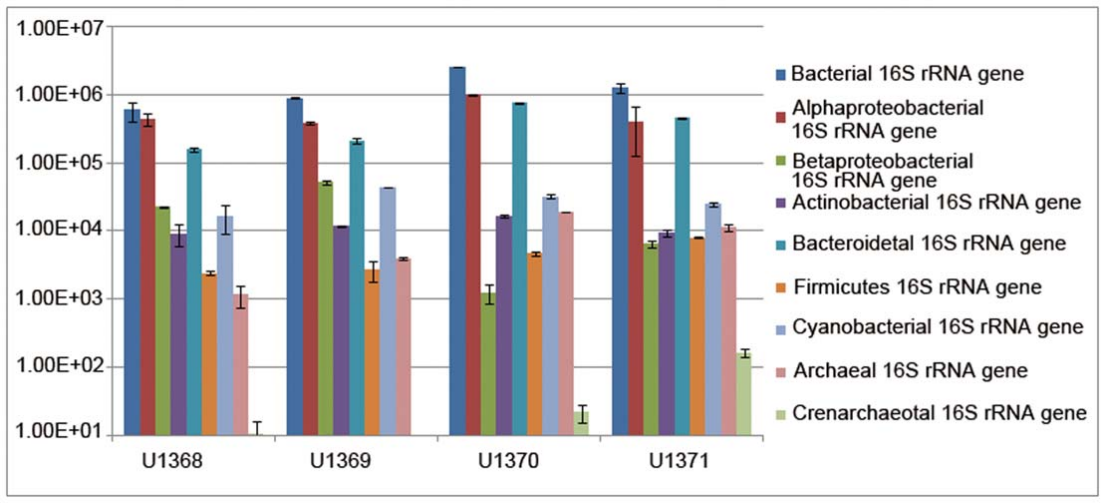
Abundances of selected microbial groups in the South Pacific Ocean surface seawaters (16S rRNA gene abundance ml ^−1^). The means and standard errors were calculated with three replicate real-time PCR measurements.

The results of real-time PCR showed that the total bacterial 16S rRNA gene abundance of four sampling sites increased gradually from the gyre center to outside, accounting for (5.96±1.87)×10^5^, (8.87±0.24)×10^5^, (2.55±0.02)×10^6^ and (1.26±0.22)×10^6^ bacterial 16S rRNA gene abundance ml^−1^ from U1368 to U1371 (Figure 7). Site U1370 showed the highest bacterial 16S rRNA gene abundance. The most abundant three groups were *Alphaproteobacteria*, *Cyanobacteria* and *Bacteroidetes*. *Firmicutes* accounted for 0.1%–0.5% in the total bacteria. *Betaproteobacteria* only accounted for 0.03% in U1370 and 0.04% in U1371. The proportion of two major bacterial groups derived from real-time PCR showed the same variation trend with the results of the clone libraries, with *Bacteroidetes* increased and *Betaproteobacteria* decreased from gyre center to edge (Figure S1). The archaeal 16S rRNA gene abundance of each site was (1.17±0.42)×10^3^ (U1368), (4.01±0.19)×10^3^ (U1369), (1.90±0.01)×10^4^ (U1370) and (1.13±0.11)×10^4^ (U1371) copies ml^−1^, respectively. The *Crenarchaeota* was only 0.001–0.015% of the total archaeal 16S rRNA gene copies.

### Statistical Comparison of Microbial Community Structures

The constitution of 16S rRNA gene sequences among the four sampling sites was statistically compared using the weighted UNIFRAC clustering method [27]. Basically, the communities from the gyre center and edge sites formed two large clusters both in bacteria and archaea. The bacterial and archaeal communities from two gyre center sites, U1368 and U1369 clustered together, while two gyre edge sites, U1370 and U1371 clustered together (Figure 4B and Figure 5B). The PCoA results consistently showed the same pattern of correlation among them (Figure S2). The microbial communities changed gradually from U1368 to U1371, including *Betaproteobacteria* and MG IIa mainly appeared in the two central sites, and *Cyanobacteria*, *Bacteroidetes* and MG IIb had high proportions in the two gyre edge sites.

**Figure 8:**
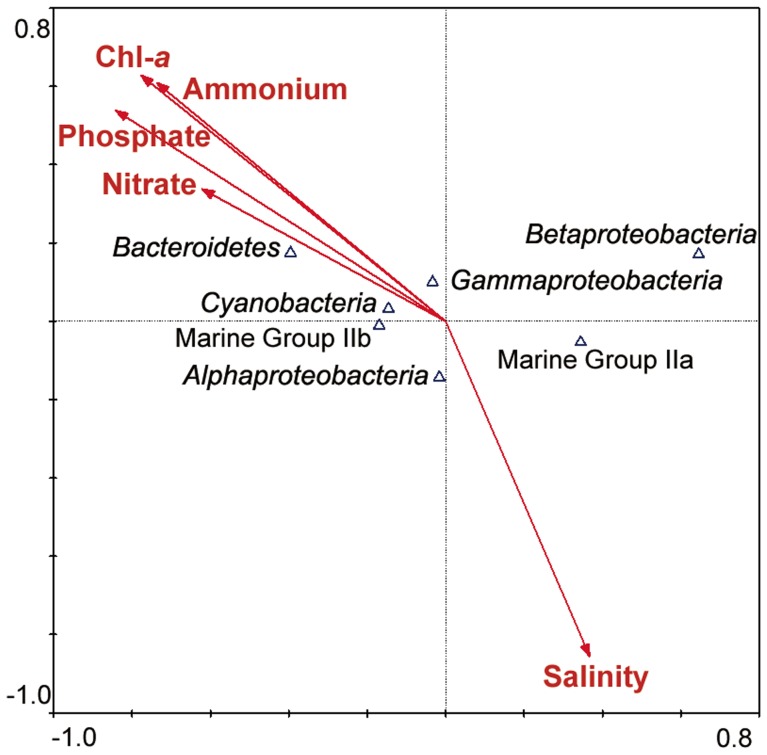
The relationship between the distribution of bacteria and archaea (7 phyla and classes) and 5 environmental factors in four sites of SPG analyzed by CCA.

CCA results of several main microbial group assemblages in response to several environmental factors were shown in Figure 8. Correlations between specific environmental factors and microbial groups were represented by the angle of arrows between them. The data indicated that geographical distribution of microbial groups from four sites was mainly influenced by the nutrient condition of the SPG. Nitrate contributed strongly in the spatial distribution of the *Cyanobacteria* and *Bacteroidetes*, while ammonia had a negative contribution to the *Gammaproteobacteria* distribution (Figure 8). On the other hand, *Betaproteobacteria* had notable negative correlation with all the mentioned nutrients, particularly with nitrate. MG IIb was positive correlation with nutrients, while MG IIa was on the opposite correlation. The dissimilarity of bacterial and archaeal community structures from different sites was measured by one-way-ANOVA in SPSS. Both the bacterial and archaeal dissimilarity results indicated that U1370 was a very different region comparing to others, because all the Tukey *P* (six in all) at bacteria and archaea level were less than 0.02 when U1370 were compared to the other three sites.

## Discussion

The SPG is the most oligotrophic ocean region in the world because of extremely low concentration of Chl-*a* and nutrients [4], [6]. The concentrations of Chl-*a*, nitrate, ammonium and phosphate of surface seawater were close to the detection limits, and had decreasing trends from the gyre edge to the center (Table 2). Previous studies have demonstrated that the rates of N_2_-fixation in the SPG and its southern edge (0.01–0.08 nM N h^−1^) [6] were substantially lower than those in the North Pacific Gyre (NPG) (0.01–0.15 nM N h^−1^) [28] and in the tropical Atlantic (up to 3.1 nM N h^−1^) [29].

In our study, it was found that the 16S rRNA gene abundance in surface seawater of the SPG were relatively low, ranging from 5.96×10^5^ to 2.55×10^6^ copies ml^−1^ at four sampling sites along the transect line from the centre to the southern edge of the gyre. This is 2 to 3 orders of magnitude lower than what has been found in eutrophic sea areas, such as the Central Baltic Sea (6.59×10^7^ to 1.62×10^9^ 16S rRNA gene copies ml^−1^) [30]. As one cell may contain multiple copies of 16S rRNA genes [30], [31], [32], the actual cell abundance should be lower than the 16S rRNA gene abundance. Indeed, it was recently reported that the microbial abundance in surface seawater of the SPG ranged from 1.5×10^5^ to 6×10^5^ cells ml^−1^ [6], which was consistent with our results, whereas the microbial abundance in surface seawater of the NPG was approximately 5.2×10^6^ cells ml^−1^ (heterotrophic bacteria only) [33], indicating that the biomass of the gyre center was much lower than other reported ocean regions. In addition, the bacterial diversity in surface seawater of the SPG was relatively low, which represents only 5 phyla (i.e., *Actinobacteria*, *Bacteroidetes*, *Cyanobacteria*, *Proteobacteria* and *Verrucomicrobia*), while the surface seawater of other sea areas harbors more diverse microbial components; e.g. the Red Sea, the Delaware coast and the NPG had 7, 8 and 7 (at least) phyla, respectively [33], [34], [35].

The microbial community composition of the SPG showed some common characteristics of the global ocean. It is now well established that *Alphaproteobacteria* (mainly represented by the *SAR11*and *SAR116* clusters) and *Cyanobacteria* (especially the *Prochlorococcus* clade) globally dominated the surface seawater [36]. In this study, they were also found dominating seawater in the SPG, accounting for the *Alphaproteobacteria* (44±11.8%; represented by the SAR11 cluster, 19±7.6% and *SAR116* cluster, 13±8.4%) and *Cyanobacteria* (25.4±5.9%; represented by *Prochlorococcus* 10.9%).

An apparent shift of microbial communities was observed from the centre to the southern edge of the gyre following the gradients of the Chl-*a* and nutrient salts (nitrate, ammonium and phosphate) concentrations. For example, 16S rRNA gene abundance of both bacteria and archaea, and the proportions of *Bacteroidetes* and MG IIb increased gradually from the center to the edge; whereas, the proportions of *Betaproteobacteria* and MG IIa decreased gradually from the center to the edge. Moreover, results of clustering relationship and PCoA by UniFrac indicate marked dissimilarities between the central gyre (U1368 & U1369) and the southern edge (U1370 & U1371). CCA results confirmed that the nutrient concentration might provide a strong barrier to community structure between the gyre center and the edge. It is noteworthy that a relatively high proportion of *Betaproteobacteria* were detected in the central gyre (accounting for 12.2% in U1368 and 19.9% in U1369), while only one clone in the U1370 and none in U1371. It has been reported that the abundance of *Betaproteobacteria* correlates strongly with salinity, and its abundance decreases as much as fourfold throughout the increase of salinity gradient [37]. However, in this study, there are no significantly changes in salinity between the gyre center and the edge. Hence, the high abundance of *Betaproteobacteria* in the gyre center may be related with the low concentrations of Chl-*a* and nutrient salts (e.g., nitrate, ammonium and phosphate). We also found that almost all members of *Betaproteobacteria* were affiliated to the genus *Achromobacter*, and its possible function in the gyre center will be discussed later on.

Due to the extremely oligotrophic features, the bacterial community composition of the SPG was also different from other pelagic ocean. Generally, *Betaproteobacteria* occurred in pelagic and surface seawater samples, however, usually at a small proportions approximately <3% [33], [36], [38], [39], [40], [41]. Nevertheless, in our study, a relatively high proportion of *Betaproteobacteria* obtained from the clone libraries was detected in the central gyre, which is distinguished from previous observations in other oceanographic sites. Compared with the less oligotrophic NPG center (HOT Station ALOHA), the SPG center site U1368 contained 12.2% of *Betaproteobacteria* and only 0.6% of *Bacteroidetes* in the bacterial library, while the NPG only had 0.34% *Betaproteobacteria* and approximate 5.1% *Bacteroidetes* [33]. Considering the predominant genera in *Alphaproteobacteria* and *Gammaproteobacteria*, SAR11 was the most dominant clade of the SPG, accounting for 43% of the *Alphaproteobacteria*, while SAR11 was the sub-dominant clade in the NPG, accounting for 11.4% of *Alphaproteobacteria* (the dominant genus was *Dechlorospirillum*, 17.2%) [33]. *Stenotrophomonas* was the predominantly detected phylotype (approximately 37.5%) within *Gammaproteobacteria* of the SPG, while it only had 0.17% in *Gammaproteobacteria* of the NPG (the dominant genus was *Glaciecola*, 28.5%). CCA results showed that distribution of major microbial groups in the SPG had strong correlations with the nutrients variation.

Regarding archaeal communities in surface seawater of the SPG, it is very interesting to find that *Euryarchaeota* (354 clones belonged to Marine Group II and 1 clone belonged to Marine Group III) dominated the four archaeal libraries, and no clones belonged to *Crenarchaeota*. However, the clonal frequencies of *Euryarchaeota* in the surface seawater of other oceans were much lower, e.g., 95% (84.5% belonged to Marine Group II) in the NPG [33] and less than 81.5% in the North Atlantic Ocean [42]. Additionally, real-time PCR results showed that the members of *Crenarchaeota* (e.g, Marine Group I) had very low frequencies (0.001%–0.015% of the total archaeal 16S rRNA gene copies), while the frequency was 5% in the NPG and 18.5% in the North Atlantic Ocean [33], [42]. *Nitrosopumilus* sp. and other Marine Group I *Crenarchaeota* are known as ammonia oxidizer [40], [43], and the very low frequency of *Crenarchaeota* 16S rRNA genes may be resulted from the low ammonium concentration in the SPG surface seawater (0.01–0.2 µM from the center to the edge) [6]. Coincidently, the rates of N_2_-fixation, which is one of the most energy-consuming metabolic pathways, were SPG<NPG<North Atlantic Ocean [6], [33], [42]. Moreover, *Euryarchaeota* shows distinct archaeal class-level lineages dominating in four sites (Table S2). While the members of MG IIa dominated the central gyre, MG IIb dominated the gyre edge. They had different relationship with the nutrients (Figure 8). Although their metabolic functions are largely unknown, these members may play important role in the carbon cycle of this ultra-oligotrophic marine ecosystem. Furthermore, nine archaeal OTUs (12 clones) in the SPG showed low similarities (94%–96%) with the known sequences, and they formed two independent clades in the phylogenetic tree (from AU1371-99 to AU1370-16 and from AU1369-36 to AU1368-84; Figure 6), which may represent new subclasses or orders of archaea.

At the genus level, four sites shared 5 known genera: “*Candidatus*” *Pelagibacter*, “*Candidatus*” *Puniceispirillum*, *Thalassobius*, *Bacillariophyta* and *Prochlorococcus*, which are all commonly observed bacteria in surface seawater. Genera had significant variations from the gyre center to the edge including *Achromobacter*, *Stenotrophomonas* and *Kordiimonas*, which had much higher abundance in the gyre center than the edge. Coincidently, members of the three genera can degrade kinds of refractory organic matters, such as polycyclic hydrocarbons [43], aromatic or halogenated compounds [44], [45], which may make them to have better chance to survive in the ultra**-**oligotrophic gyre center. In addition, some of the *Achromobacter* spp. can perform nitrate respiration [43] and ammonia assimilation [37], whereas *Stenotrophomonas* spp. are free-living nitrogen fixing bacteria [46], both of which may play a role in the nitrogen cycle of this ultra-oligotrophic marine ecosystem.

In this study, we have also cultivated some aerobic heterotrophic bacteria from the surface seawater of the SPG. The percentage of novel bacterial strains was relatively high. Among 74 strains of the cultivated bacteria, 14 strains (18.9%) were potentially novel bacteria, affiliating to *Flavobacteria* (9 strains), *Alphaproteobacteria* (2 strains), and *Gammaproteobacteria* (3 strains), based on 16S rRNA gene sequence of the isolates. It was found that the percentages of potentially novel bacterial strains in the cultivated bacteria were 8.5% in the open Pacific sites and 5.9% in the Peru Margin sites of subseafloor sediments in eastern Pacific Ocean [47]. The sequences of *Thalassobius* spp. were found both in the clone libraries (U1368-4) and the cultivated bacteria (SW101). In addition, SW177 from the cultivated bacteria and U1369-152 from the clone libraries clustered together.

In conclusion, the microbial communities in surface seawater of the SPG are unique and different to those reported from other oceanographic settings. The 16S rRNA gene abundance of both bacteria and archaea were much lower than organic-rich oceans, and the gene abundance increased gradually from the center to the edge of the gyre. Only 5 phyla were observed as major bacterial component in the clone libraries, and *Betaproteobacteria* had a relatively high proportion in the centre of the gyre as compared with other ocean area. For archaeal components, unlike most other surface seawater, the members of Marine Group II within the *Euryarchaeota* almost dominated the whole archaea group in the examined SPG samples, while no clones of 16S rRNA genes within the *Crenarchaeota* were obtained. Some of the cultivated bacteria might have ecologically important metabolic pathways, which is worthy of more research. Further and detailed survey, e.g. high-throughput sequencing of the SPG seawater samples will reveal complete compositions of the ultra-oligotrophic microbial communities.

## Supporting Information

Figure S1 **S1 PCoA results showing the relatedness of (a) bacterial and (b) archaeal communities in the surface seawater of four stations in SPG.** The PCoA plots were constructed with the weighted UniFrac PCoA method.
(TIF)

Figure S2 **The same variation trend of two main bacterial groups in clone library and qPCR from gyre center to edge, with** ***Bacteroidetes*** **increased and** ***Betaproteobacteira*** **decreased.**
(TIF)

Table S1 **Classification of bacterial clones at each taxonomic level for the four surface seawater communities in SPG, based on the blast results of RDP classifer and EzTaxon server 2.1.**
(DOCX)

Table S2 **Classification of archaeal clones at each taxonomic level for the four surface seawater communities in SPG, based on the blast results of RDP classifer and EzTaxon server 2.1.**
(DOCX)
