# Supplementary figures and images for: Spatial Variations in Microbial Community Composition in Surface Seawater from the Ultra-Oligotrophic Center to Rim of the South Pacific Gyre
Source: PLoS One. 2013 Feb 6;8(2):e55148. doi: 10.1371/journal.pone.0055148 (PMC3566182; doi:10.1371/journal.pone.0055148)

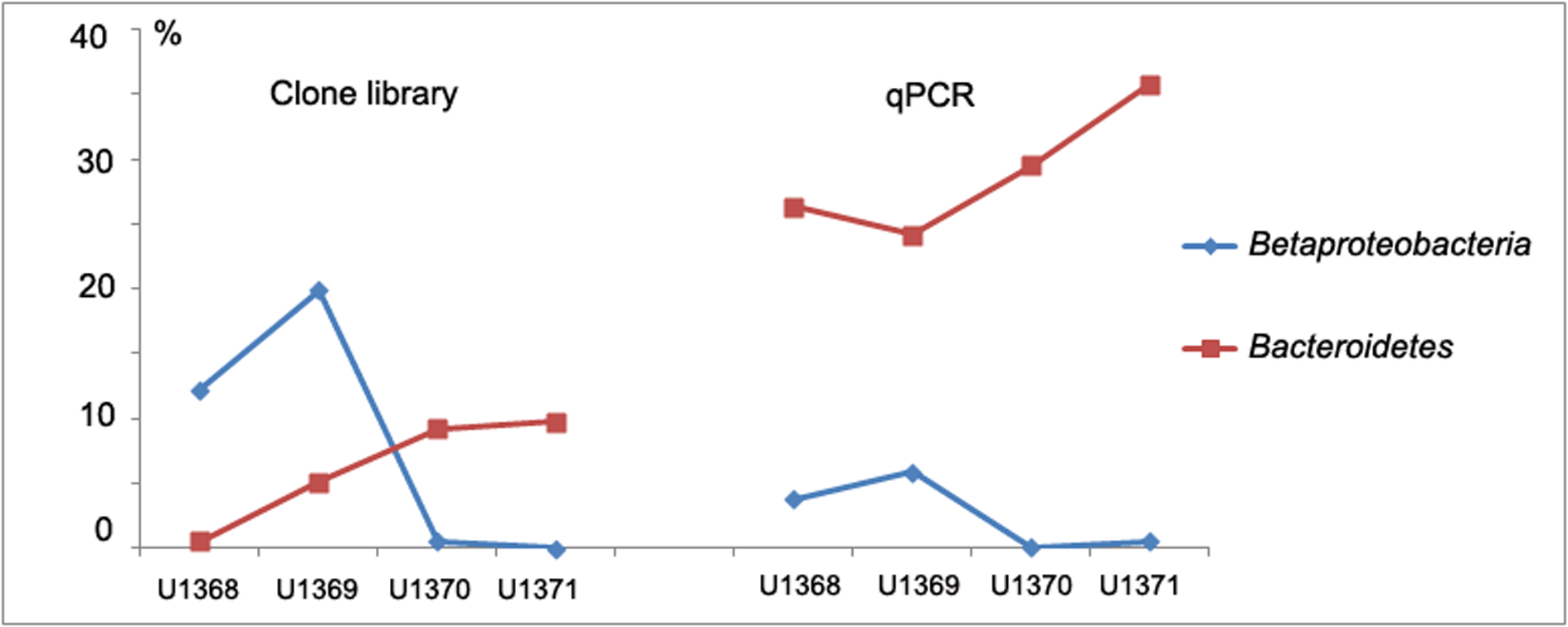

Supplement: Figure S1 — S1 PCoA results showing the relatedness of (a) bacterial and (b) archaeal communities in the surface seawater of four stations in SPG. The PCoA plots were constructed with the weighted UniFrac PCoA method. (TIF) [file pone.0055148.s001.tif]

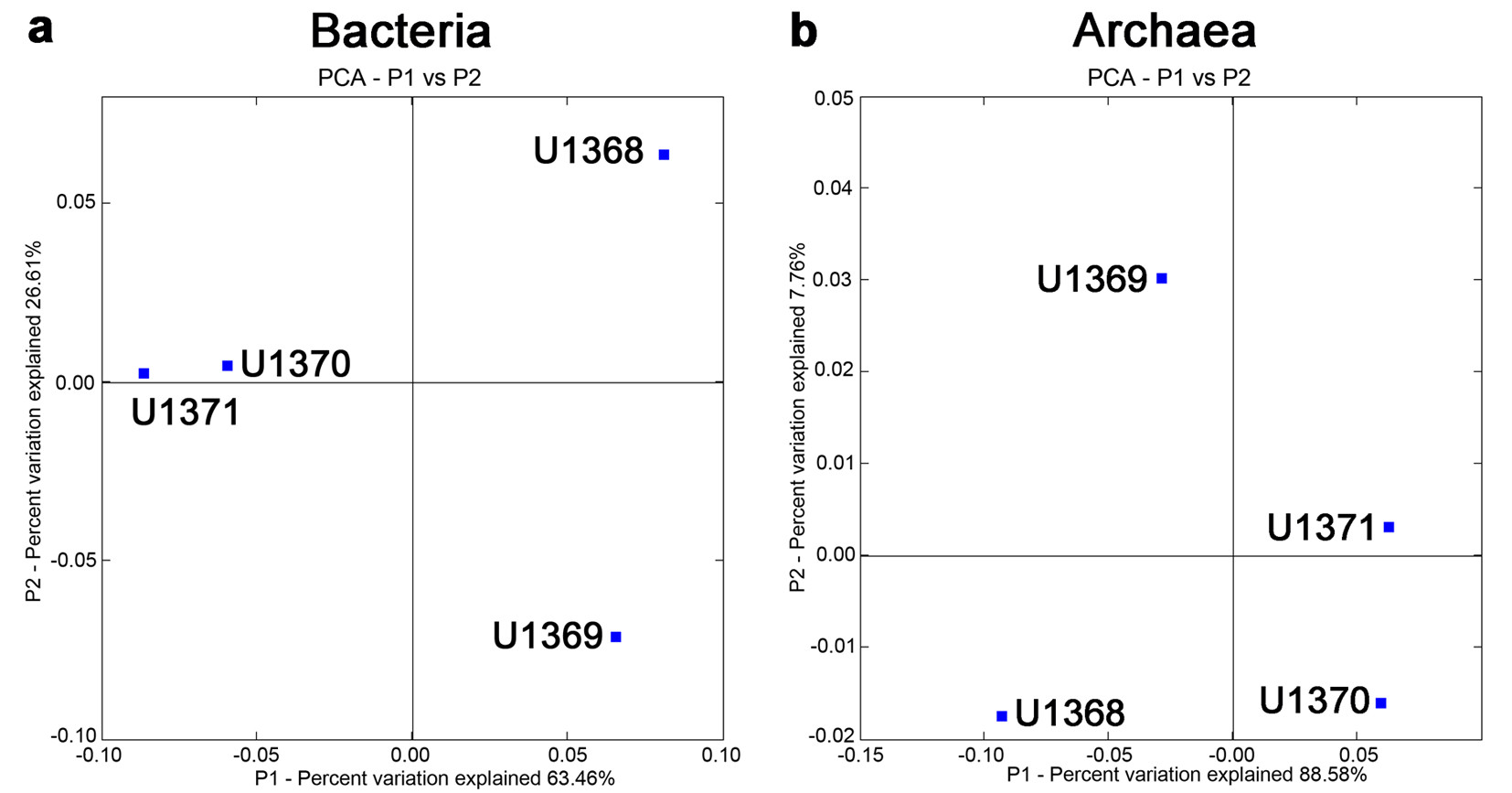

Supplement: Figure S2 — The same variation trend of two main bacterial groups in clone library and qPCR from gyre center to edge, with Bacteroidetes increased and Betaproteobacteira decreased. (TIF) [file pone.0055148.s002.tif]
