# Supplementary material for: Spatial Variations in Microbial Community Composition in Surface Seawater from the Ultra-Oligotrophic Center to Rim of the South Pacific Gyre
Source: PLoS One. 2013 Feb 6;8(2):e55148. doi: 10.1371/journal.pone.0055148 (PMC3566182; doi:10.1371/journal.pone.0055148)
Supplement: Table S1 — Classification of bacterial clones at each taxonomic level for the four surface seawater communities in SPG, based on the blast results of RDP classifer and EzTaxon server 2.1. (DOCX) [file pone.0055148.s003.docx]

Table S1 Classification of bacterial clones at each taxonomic level for the four surface seawater communities in SPG, based on the blast results of RDP classifer and EzTaxon server 2.1.

|  | U1368 | U1369 | U1370 | U1371 |
| --- | --- | --- | --- | --- |
| **Domain** | 180 | 196 | 196 | 185 |
| unclassified | 0 | 1 | 6 | 3 |
| Bacteria | 180 | 195 | 190 | 182 |
| **Phylum** |  |  |  |  |
| unclassified | 0 | 1 | 6 | 3 |
| Proteobacteria | 146 | 136 | 110 | 101 |
| Bacteroidetes | 1 | 10 | 18 | 18 |
| Cyanobacteria | 29 | 48 | 59 | 57 |
| Actinobacteria | 2 | 1 | 3 | 1 |
| Verrucomicrobia | 2 | 0 | 0 | 5 |
| **Class** |  |  |  |  |
| unclassified | 0 | 1 | 6 | 3 |
| Alphaproteobacteria | 107 | 61 | 89 | 74 |
| Gammaproteobacteria | 16 | 34 | 20 | 27 |
| Betaproteobacteria | 22 | 39 | 1 | 0 |
| Deltaproteobacteria | 1 | 2 | 0 | 0 |
| unclassified Bacteroidetes | 1 | 7 | 16 | 9 |
| Flavobacteria | 0 | 3 | 2 | 8 |
| Cytophagia | 0 | 0 | 0 | 1 |
| Cyanobacteria | 29 | 48 | 59 | 57 |
| Actinobacteria | 2 | 1 | 3 | 1 |
| unclassified Verrucomicrobia | 1 | 0 | 0 | 5 |
| Opitutae | 1 | 0 | 0 | 0 |
| **Order** |  |  |  |  |
| unclassified | 0 | 1 | 6 | 3 |
| unclassified Alhpaproteobacteria | 3 | 4 | 7 | 4 |
| Rickettsides | 39 | 15 | 40 | 52 |
| Rhodospirillales | 30 | 12 | 18 | 4 |
| Rhodobacterales | 20 | 27 | 16 | 12 |
| Rhizobiales | 0 | 0 | 8 | 2 |
| Kordiimonadales | 15 | 3 | 0 | 0 |
| unclassified Gammaproteobacteria | 6 | 10 | 12 | 14 |
| Aeromonasales | 0 | 0 | 0 | 7 |
| Porticoccus | 0 | 1 | 0 | 2 |
| Candidatus | 2 | 1 | 4 | 2 |
| Pseudomonadales | 0 | 0 | 0 | 1 |
| Oceanospirillales | 2 | 1 | 1 | 1 |
| Chromatiales | 0 | 0 | 3 | 0 |
| Xanthomomadales | 6 | 21 | 0 | 0 |
| Methylophilales | 0 | 1 | 1 | 0 |
| Burkholderiales | 22 | 38 | 0 | 0 |
| Desulfuromonadales | 1 | 1 | 0 | 0 |
| Desulfobacterales | 0 | 1 | 0 | 0 |
| unclassified Bacteroidetes | 1 | 7 | 16 | 9 |
| Flavobacteriales | 0 | 3 | 2 | 8 |
| Cytophagales | 0 | 0 | 0 | 1 |
| Cyanobacteria | 22 | 37 | 57 | 53 |
| Prochlorales | 7 | 11 | 2 | 4 |
| unclassified Actinobacteria | 2 | 1 | 3 | 1 |
| unclassified Verrucomicrobia | 2 | 0 | 0 | 5 |
| **Family** |  |  |  |  |
| unclassified | 34 | 48 | 76 | 53 |
| unclassified Rickettsides | 0 | 0 | 1 | 0 |
| SAR11 | 39 | 15 | 40 | 46 |
| SAR116 | 24 | 10 | 14 | 4 |
| Rhodospirillaceae | 6 | 2 | 4 | 0 |
| Rhodobacteraceae | 20 | 27 | 16 | 12 |
| Phyllobacteriaceae | 0 | 0 | 3 | 2 |
| Hyphomicrobiaceae | 0 | 0 | 5 | 0 |
| Kordiimonadaceae | 15 | 3 | 0 | 0 |
| unclassified Pseudomonadales | 0 | 0 | 0 | 1 |
| unclassified Chromatiales | 0 | 0 | 3 | 0 |
| Proticoccus | 0 | 1 | 0 | 2 |
| Aeromonadaceae | 0 | 0 | 0 | 7 |
| BermanellaA | 0 | 0 | 1 | 0 |
| Oleiphilaceae | 0 | 1 | 0 | 1 |
| Thiobios | 2 | 1 | 4 | 2 |
| Xanthomomadaceae | 6 | 21 | 0 | 0 |
| Methylophilaceae | 0 | 1 | 1 | 0 |
| Alcaligenaceae | 22 | 38 | 0 | 0 |
| Geobacteraceae | 1 | 1 | 0 | 0 |
| Desulfbulbaceae | 0 | 1 | 0 | 0 |
| unclassified Flavobacteriales | 0 | 1 | 2 | 2 |
| Cryomorphaceae | 0 | 1 | 0 | 3 |
| Flavobacteriaceae | 0 | 1 | 0 | 3 |
| Cytophagaceae | 0 | 0 | 0 | 1 |
| Chloroplast | 3 | 11 | 24 | 37 |
| Prochlorococcaceae | 7 | 11 | 2 | 4 |
| unclassified Verrucomicrobiales | 0 | 0 | 0 | 5 |
| Puniceicoccaceae | 1 | 0 | 0 | 0 |
| **Genus** |  |  |  |  |
| unclassified | 36 | 50 | 80 | 75 |
| Candidatus Pelagibacter | 39 | 15 | 39 | 46 |
| Candidatus Puniceispirillum | 24 | 10 | 14 | 4 |
| Oceanibaculum | 4 | 1 | 2 | 0 |
| unclassified Rhodospirillaceae | 2 | 1 | 2 | 0 |
| unclassified Rhodobacteraceae | 3 | 3 | 2 | 1 |
| Nitratireductor | 0 | 0 | 3 | 2 |
| Zhangella | 0 | 0 | 5 | 0 |
| Kordiimonas | 15 | 3 | 0 | 0 |
| Thalassobius | 7 | 13 | 8 | 7 |
| Pacificibacter | 0 | 1 | 1 | 1 |
| Ruegeria | 4 | 0 | 0 | 0 |
| Pseudoruegeria | 1 | 0 | 1 | 0 |
| Sulfitobacter | 0 | 5 | 0 | 0 |
| Octadecabacter | 0 | 0 | 0 | 1 |
| Roseovarius | 1 | 0 | 0 | 0 |
| Roseobacter | 0 | 0 | 1 | 0 |
| Tropicibacter | 0 | 1 | 1 | 0 |
| Pelagicola | 2 | 4 | 0 | 0 |
| unclassified Porticoccus | 0 | 1 | 0 | 2 |
| unclassified Bermanella | 0 | 0 | 1 | 0 |
| Oleiphilus | 0 | 1 | 0 | 1 |
| unclassified Thiobios | 2 | 1 | 4 | 2 |
| Stenotrophomonas | 6 | 21 | 0 | 0 |
| unclassifed Methylophilaceae | 0 | 1 | 1 | 0 |
| Achromobacter | 22 | 38 | 0 | 0 |
| Geobacter | 1 | 1 | 0 | 0 |
| Desulfobulbus | 0 | 1 | 0 | 0 |
| unclassified Cryomorphaceae | 0 | 1 | 0 | 3 |
| unclassified Cytophagaceae | 0 | 0 | 0 | 1 |
| unclassified Flavobacteriaceae | 0 | 1 | 0 | 2 |
| Bizionia | 0 | 0 | 0 | 1 |
| Unclassified Chloroplast | 0 | 6 | 11 | 7 |
| Bacillariophyta | 3 | 5 | 13 | 30 |
| Prochlorococcus | 7 | 11 | 2 | 4 |
| Coraliomargarita | 1 | 0 | 0 | 0 |
|  | | | | |
