# Supplementary material for: Spatial Variations in Microbial Community Composition in Surface Seawater from the Ultra-Oligotrophic Center to Rim of the South Pacific Gyre
Source: PLoS One. 2013 Feb 6;8(2):e55148. doi: 10.1371/journal.pone.0055148 (PMC3566182; doi:10.1371/journal.pone.0055148)
Supplement: Table S2 — Classification of archaeal clones at each taxonomic level for the four surface seawater communities in SPG, based on the blast results of RDP classifer and EzTaxon server 2.1. (DOCX) [file pone.0055148.s004.docx]

Table S2 Classification of archaeal clones at each taxonomic level for the four surface seawater communities in SPG, based on the blast results of RDP classifer and EzTaxon server 2.1.

|  | U1368 | U1369 | U1370 | U1371 |
| --- | --- | --- | --- | --- |
| **Domain** | 180 | 196 | 196 | 185 |

| Archaea | 88 | 92 | 88 | 87 |
| --- | --- | --- | --- | --- |
| **Phylum** |  |  |  |  |
| Euryarchaeota | 88 | 92 | 88 | 87 |
| **Class** |  |  |  |  |
| Marine Group II |  |  |  |  |
| MG IIa | 47 | 40 | 9 | 10 |
| MG IIb | 41 | 52 | 78 | 77 |
| Marine Group III |  |  |  |  |
| Thermoplasmata | 0 | 0 | 1 | 0 |
